# Supplementary figures and images for: CytoBinning: Immunological insights from multi-dimensional data
Source: PLoS One. 2018 Oct 31;13(10):e0205291. doi: 10.1371/journal.pone.0205291 (PMC6209166; doi:10.1371/journal.pone.0205291)

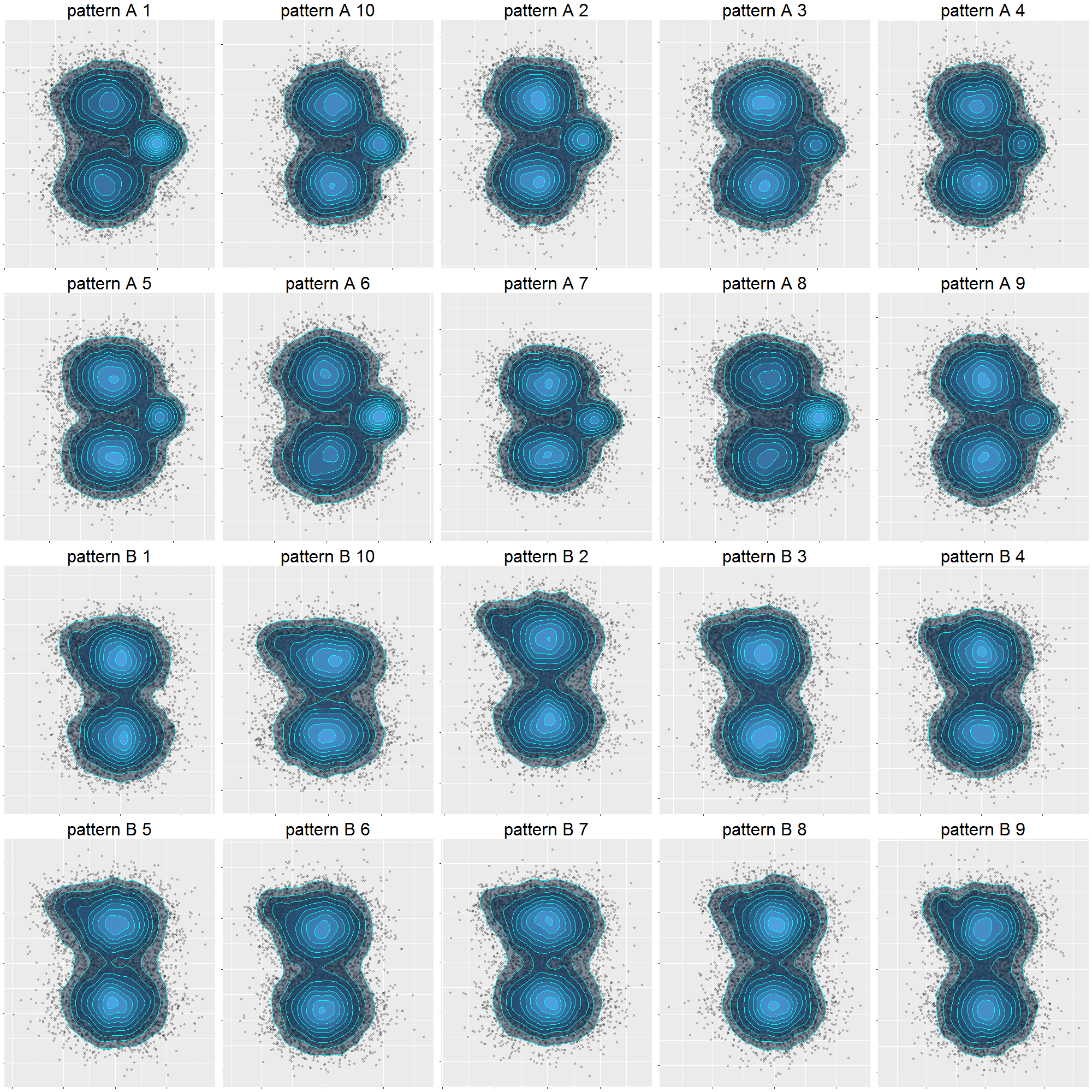

Supplement: S1 Fig — First two rows show point pattern A, the lower two rows show point pattern B. Two major clusters in both point pattern A and B are generated from the same distributions. The third cluster of point pattern A, located on center right, consists about 10 to 20% of total cells. The third cluster of point pattern B, located at upper left of all points, contains only 2 to 5% of all cells. (TIF) [file pone.0205291.s001.tif]

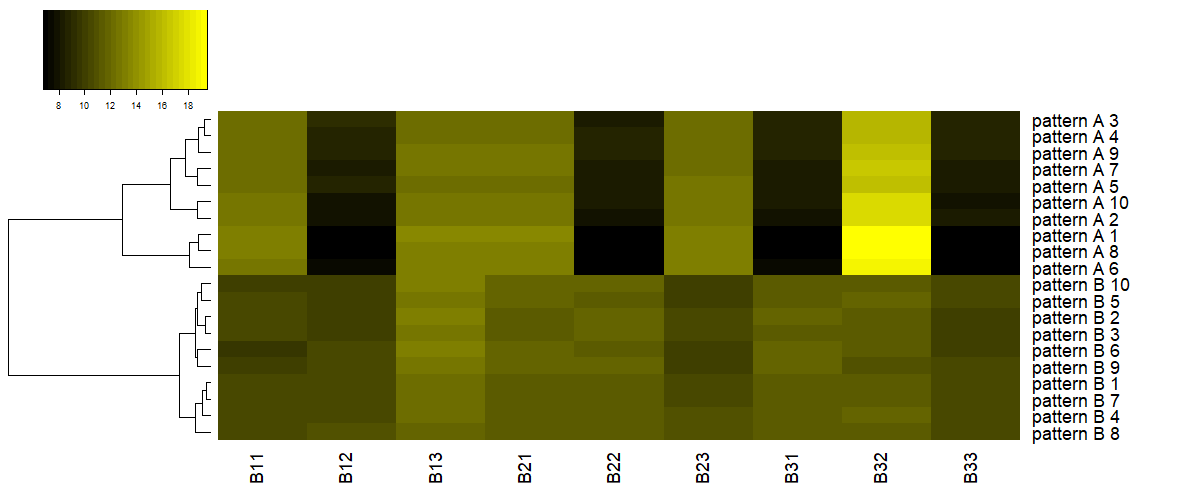

Supplement: S2 Fig — Percentage of cells in box B16 (which corresponds to the third cluster in point pattern B) is significantly different (adjusted p value = 0.0004) between these two point patterns. This is not seen with only 3 bins. However, with 20 samples, analysis results using 6 bins is not reliable. Hence, in order to identify fine difference, more samples are needed. (TIF) [file pone.0205291.s002.tif]

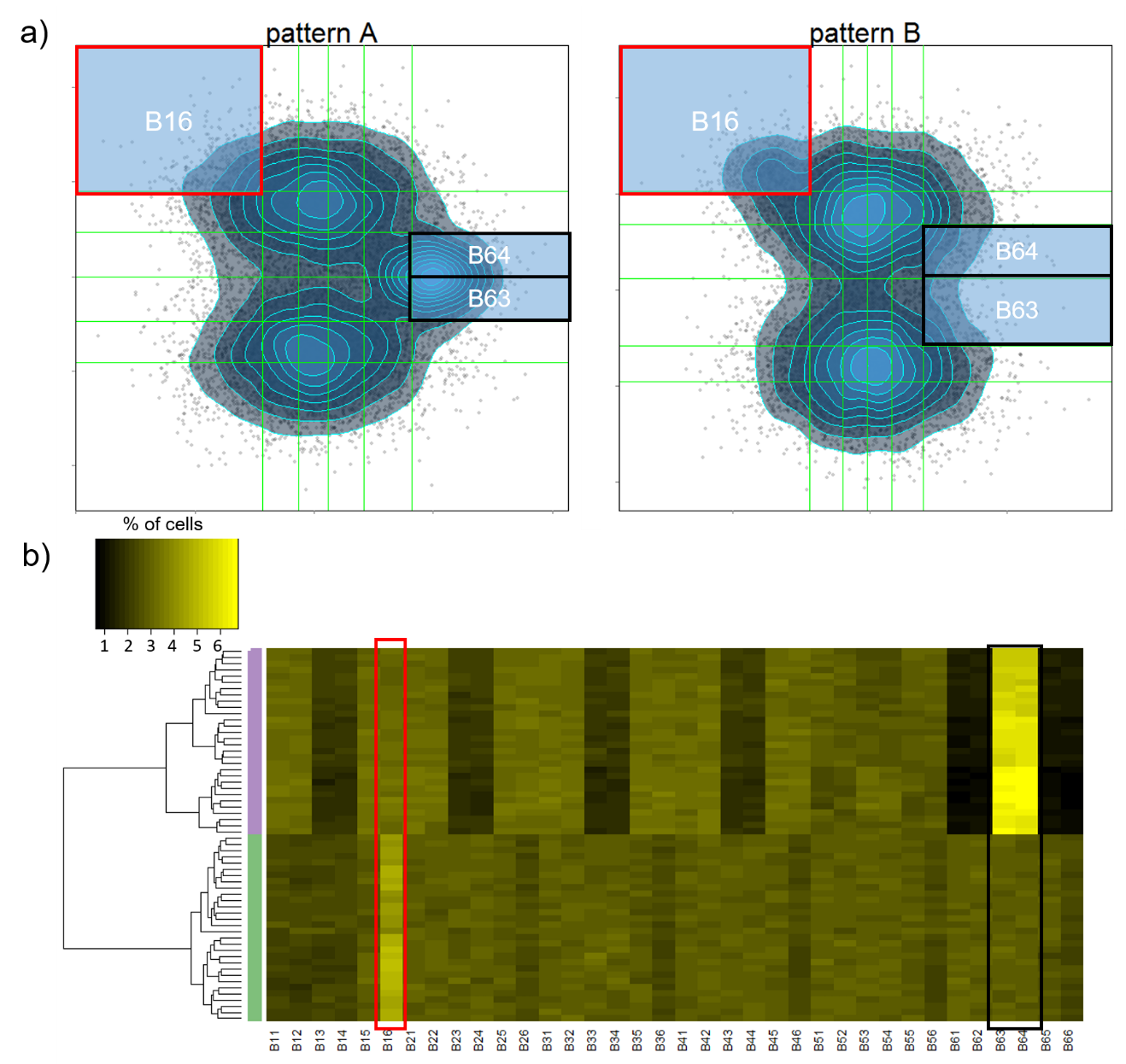

Supplement: S3 Fig — a) Example for both pattern A and pattern B. b) Heatmap showing hierarchical clustering for CytoBinning results with 6 bins. Highlighted are the most different boxes between pattern A and B (adjusted p value << 0.001 for all three boxes). (TIF) [file pone.0205291.s003.tif]

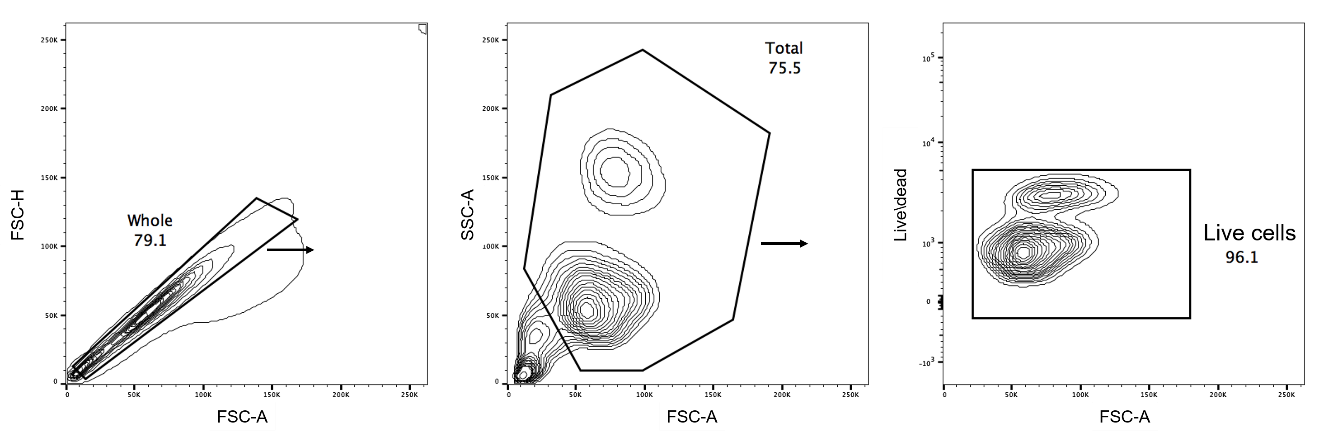

Supplement: S4 Fig — (TIF) [file pone.0205291.s004.tif]

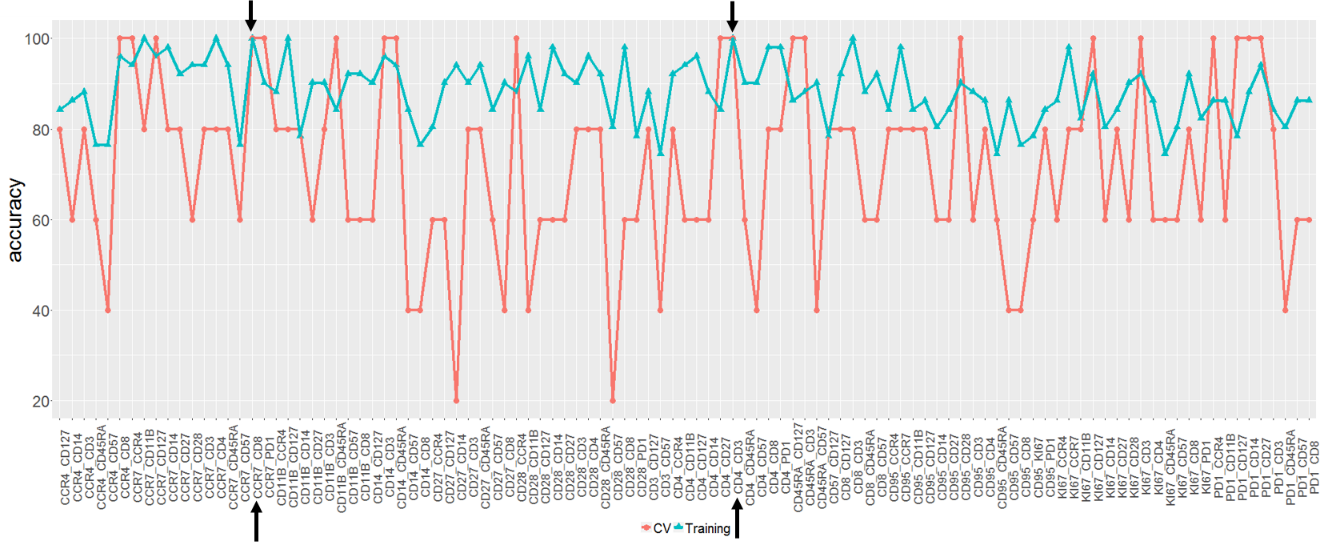

Supplement: S5 Fig — Ten samples are randomly selected as cross validation dataset (4 in young group and 6 in old group). SVM classification was used to separate old and young samples with binning results for each marker pair separately. Two marker pairs are able to achieve 100% classification accuracy for both trainning and cross validation dataset (CD4 vs CD3 and CD8 vs CCR7). (TIF) [file pone.0205291.s005.tif]

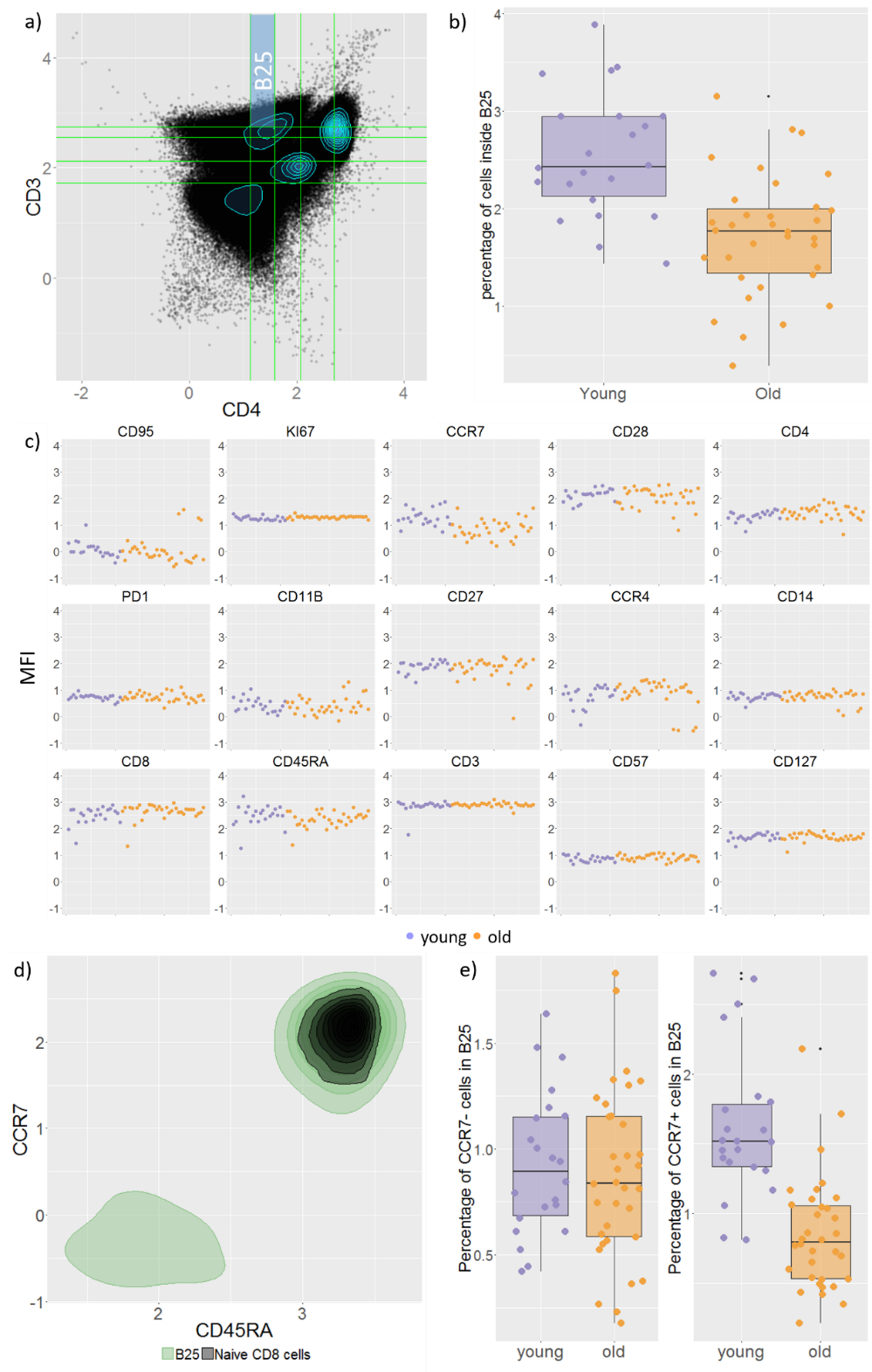

Supplement: S6 Fig — a) Position of box B25. b) Percentage of cells in B25 is higher in young donors (adjusted p value = 0.03). c) Scatter plot of mean flourescent intensity (MFI) for all donors and all markers. This suggests cells in B25 are CD3+, CD8+ and CD45RA+. d) An example showing how cells in B25 (green) compare to manually gated naïve CD8 cells. e) Cells in B25 are divided into two groups: CCR7+ (expression of CCR7>1) and CCR7- (expression of CCR7<1). The boxplots show that difference of cell percentage between old and young donors in B25 is driven by CCR7+ cells (p value << 0.001). (TIF) [file pone.0205291.s006.tif]

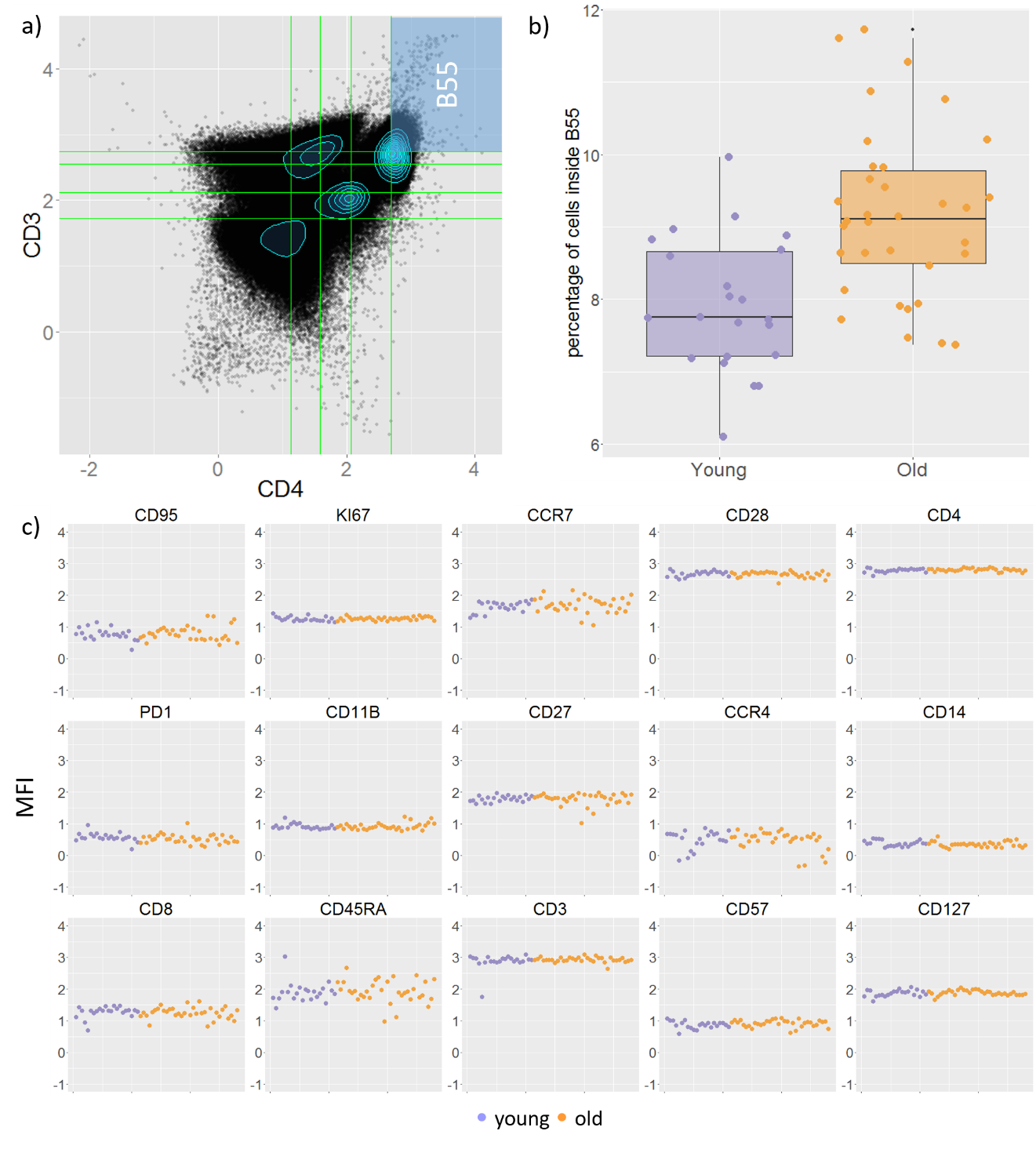

Supplement: S7 Fig — a) Position of box B55. Cells in B55 express the highest 20% of both CD3 and CD4. Hence they might be CD4 T cells. b) Percentage of cells in B55 is higher in old donors (adjusted p value = 0.05). c) Scatter plot of mean flourescent intensity (MFI) for all donors and all markers. It suggests cells in B55 might be CD8-, CCR7+ and CD45RA+. (TIF) [file pone.0205291.s007.tif]

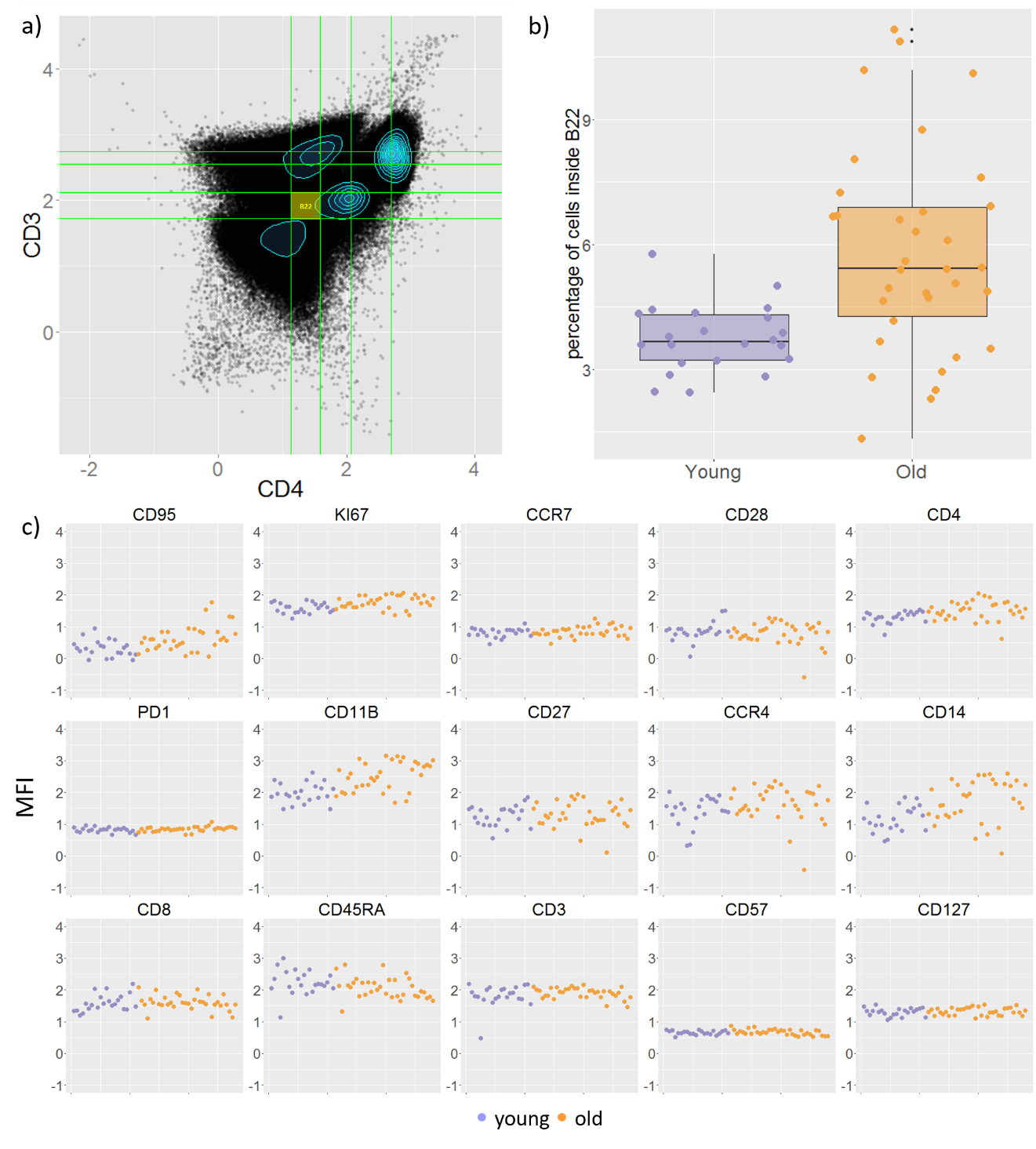

Supplement: S8 Fig — a) Position of box B22. b) Percentage of cells in B55 is higher in old donors (adjusted p value = 0.02). c) Scatter plot of mean flourescent intensity (MFI) for all donors and all markers. It suggests cells in B22 might be CD11b+, CD14+ and CD45RA+. (TIF) [file pone.0205291.s008.tif]

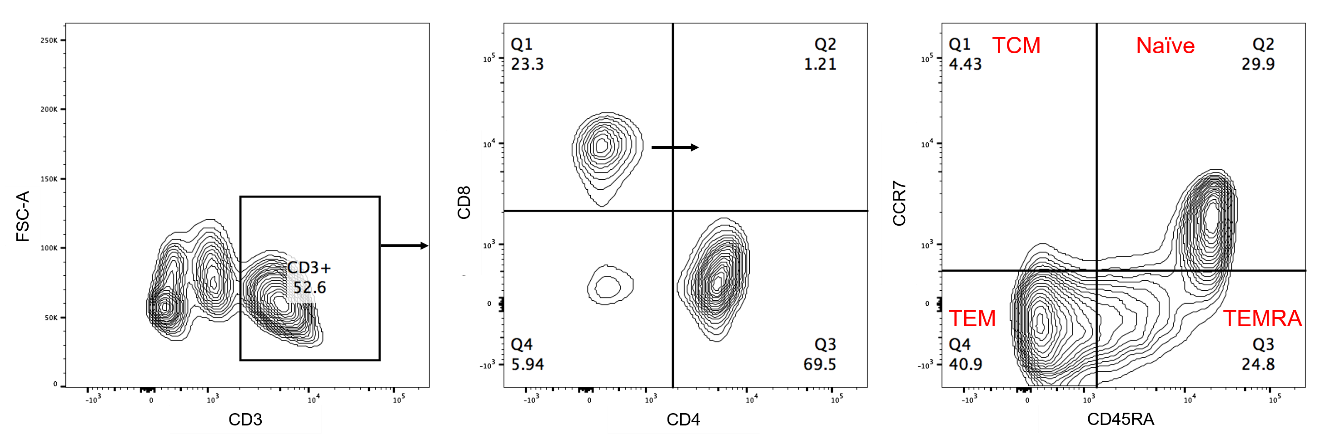

Supplement: S9 Fig — (TIF) [file pone.0205291.s009.tif]

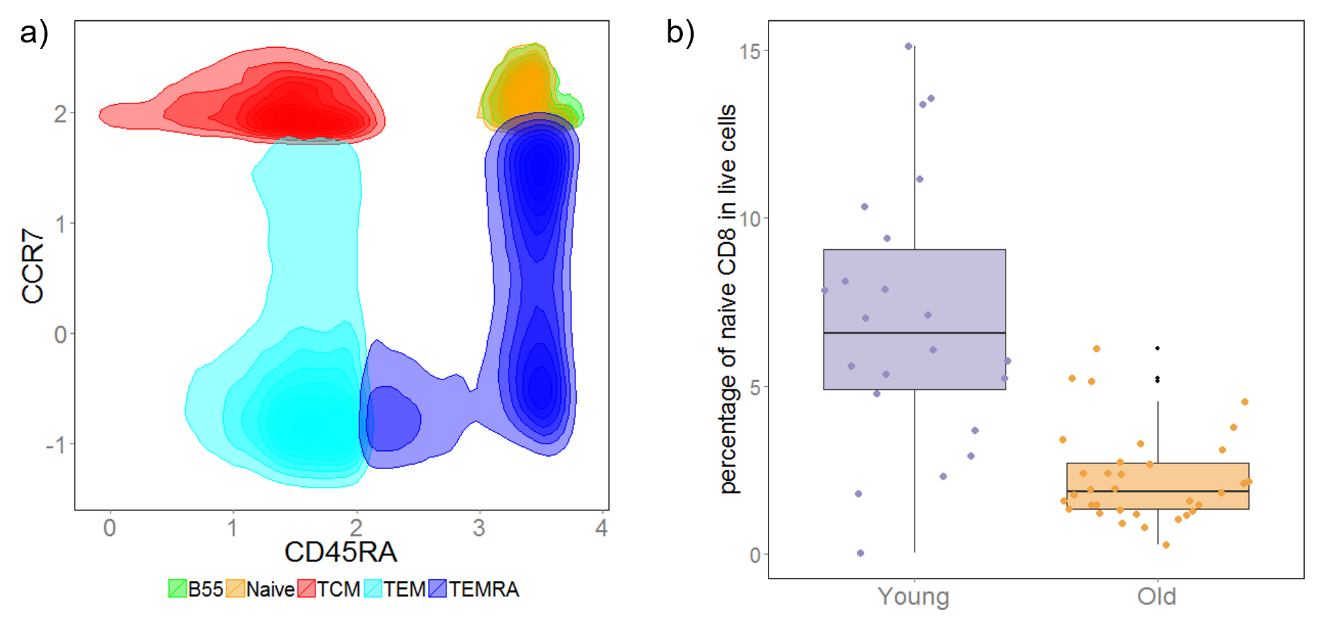

Supplement: S10 Fig — a) Overlay of cells in B55 on manually gated CD8 naïve and memory cell types for one donor. b) Boxplot of manually gated naïve CD8 cell percentage in live cells. (TIF) [file pone.0205291.s010.tif]

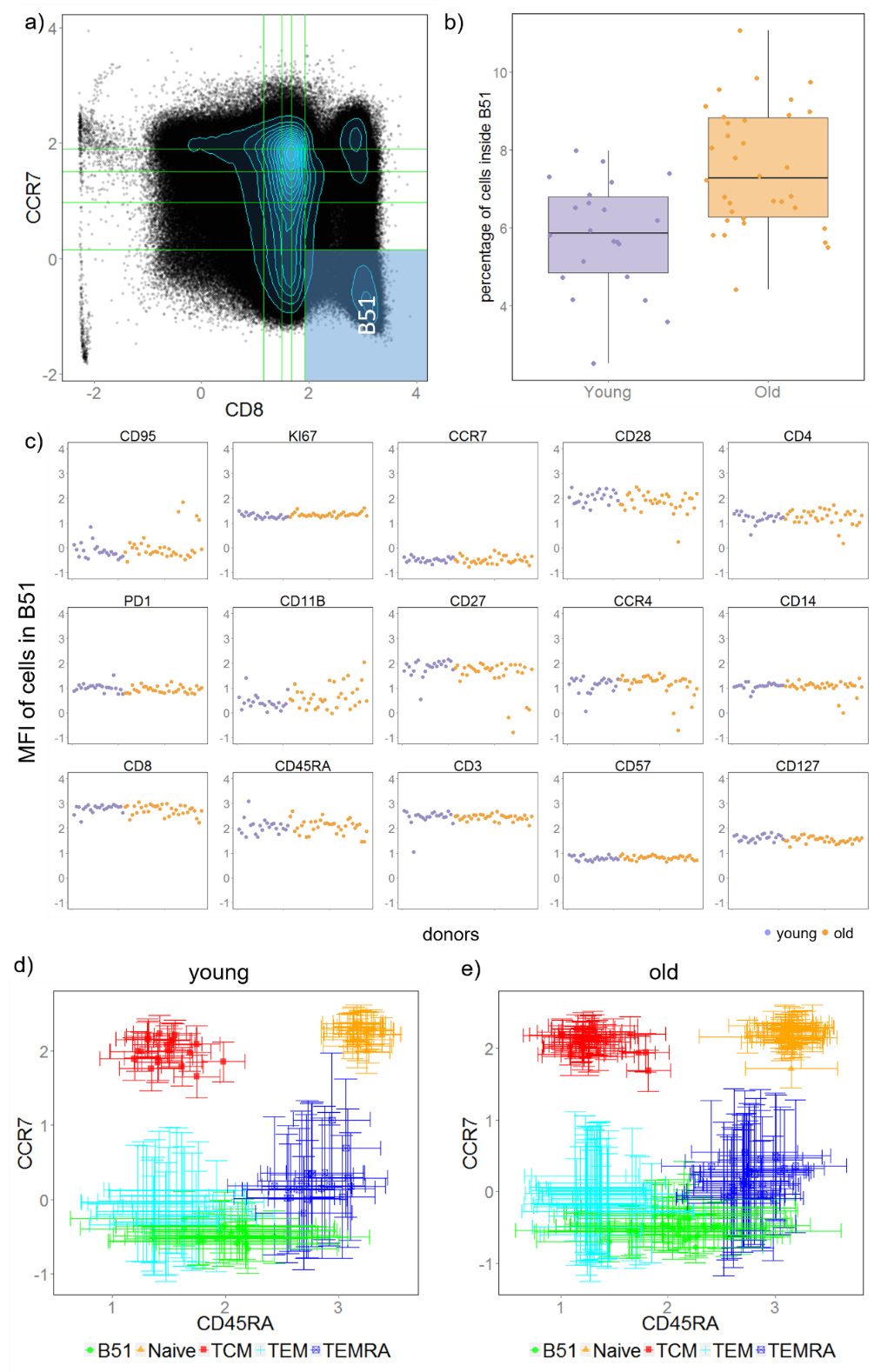

Supplement: S11 Fig — a) Position of box B51. b) Percentage of cells in B51 increased in old donors (adjusted p value = 0.01). c) Scatter plot of mean flourescent intensity (MFI) for all donors and all markers. d & e) MFI of CD45RA vs MFI of CCR7 for cells in B51, naïve and memroy CD8 T cells. Each symbol shows a donor (young donors in d and old donors in e), vertical and horizontal errorbars show standard deviation of CCR7 and CD45RA intensity respectively. (TIF) [file pone.0205291.s011.tif]
