# Supplementary material for: CytoBinning: Immunological insights from multi-dimensional data
Source: PLoS One. 2018 Oct 31;13(10):e0205291. doi: 10.1371/journal.pone.0205291 (PMC6209166; doi:10.1371/journal.pone.0205291)
Supplement: S1 List — (PDF) [file pone.0205291.s012.pdf]

S1 List. Markers measured in CD4 vs CD8 dataset

CD45, CD14, CD57, TCRgD, IFNg, TNFa, IL.8, GranzymeB, IL17F, CD45RA, CLA, CTLA.4, IL2, CD25, CD103, CCR10, CXCR6, IL5, CD19, CD56, IntegrinB7, PD1, IL9, CCR9, CXCR3, CD127, Mip1b, CXCR5, CD161, CCR2, IL4, IL10, CCR6, GM.CSF, CCR4, IL22, CCR5, IL17A
